# Notch1 regulates the functional contribution of RhoC to cervical carcinoma progression

**DOI:** 10.1038/sj.bjc.6605451

**Published:** 2009-12-01

**Authors:** S Srivastava, B Ramdass, S Nagarajan, M Rehman, G Mukherjee, S Krishna

**Affiliations:** 1 National Centre for Biological Sciences, TIFR, Bangalore, Karnataka, India; 2 Department of Pathology, Kidwai Memorial Institute of Oncology, Bangalore, Karnataka, India

**Keywords:** Notch1, RhoC, cervical carcinoma, angiogenesis, invasion, PI3K/Akt

## Abstract

**Background:**

The role of Notch signalling in human epithelial cancers is of immense interest. In this study, we examine the interplay between Notch signalling and RhoC, a well-established molecular factor in metastasis. By linking the function of Notch and RhoC, we further strengthen the notion that there is a pro-oncogenic role of Notch signalling in human cervical cancers.

**Methods:**

RhoC protein expression in cervical carcinoma cell lines was assessed by western blotting. Using CaSki and SiHa cells (cervical carcinoma cells lines), we show that RhoC contributes to wound healing, invasion and migration, anoikis resistance, colony formation, *in vitro* tube formation and tumour formation. Immunohistochemical studies were carried out to assess the co-expression of RhoC, pAkt and Notch1 in clinical sections.

**Results:**

An assessment of the changes associated with epithelial-to-mesenchymal transition (EMT) shows that both Notch1 and RhoC have similar phenotypic contribution to EMT. Rho activity assessment on Notch1 inhibition with DAPT shows decreased RhoC activity. We further show that constitutively active RhoC rescues the phenotypic effect of Notch1 inactivation, and a comparison of Notch1 with RhoC expression shows an overlap between the two proteins in the same areas of the tissue.

**Conclusion:**

This study has provided evidence to suggest that RhoC is an effector of Notch1 in cervical carcinoma.

Notch has an important role in cell fate determination, stem cell maintenance and development (Artavanis-Tsakonas *et al*, 1999). Notch1 signalling regulates the maintenance of haematopoietic stem cells and the development of T cells (Allman *et al*, 2002). The Notch receptor is activated on DSL ligand interaction-induced proteolytic cleavage, leading to the formation of intracellular Notch1 (ICN). Intracellular Notch1 translocates to the nucleus and acts as a transcriptional modulator (Honjo, 1996). Activating mutations have been reported in human T-ALL Notch1 (Weng *et al*, 2004); however, limited sequencing of a similar region in Notch1 alleles of cervical carcinoma suggests the absence of such a mutation (D Subramanyam and S Krishna, unpublished data). Dysregulated Notch signalling has been implicated in other tumours including mammary and cervical cancer (Zagouras *et al*, 1995; Daniel *et al*, 1997; Politi *et al*, 2004). Cervical carcinoma, the second most prevalent cancer in women, is initiated and sustained in part by the presence of high-risk human papillomavirus oncogene expression (zur Hausen, 2002), and has an aberrant expression of Notch1, its ligand Jagged1 and downstream target Hes1 (Ramdass *et al*, 2007).

There is a wealth of data suggesting the contribution of ICN to features of tumour progression, including invasion, epithelial-to-mesenchymal transition (EMT), metastasis and angiogenesis (Timmerman *et al*, 2004; Wang *et al*, 2006, 2007; Zhang *et al*, 2008). Notch1 induces anoikis resistance, inhibits p53 activity and upregulates myc in cervical carcinoma (Rangarajan *et al*, 2001; Nair *et al*, 2003; Klinakis *et al*, 2006; Subramanyam and Krishna, 2006; Weng *et al*, 2006) and also inhibits the growth inhibitory effects of TGF-*β* during cell growth (Masuda *et al*, 2005). Our earlier report suggests that PI3K-mediated EMT in cervical cancer correlates with Jagged1 expression (Veeraraghavalu *et al*, 2005). Although there is data supporting the role of Notch1 in tumour progression, there is paucity of evidence of factors downstream of it that regulate these processes. There is emerging evidence that RhoC regulates features of tumour progression. It regulates invasion, metastasis, EMT and angiogenesis in various carcinomas (Sequeira *et al*, 2008; Wang *et al*, 2008; Boone *et al*, 2009), regulating features similar to Notch1.

RhoC, a small GTPase, belongs to the Rho GTPase family. Activated Rho GTPases affect actin cytoskeleton dynamics, transcriptional regulation, cell cycle progression, membrane trafficking and tumour progression (Vega and Ridley, 2008). It has also been shown that functional Rho GTPases are required for Ras-dependent transformation (Qiu *et al*, 1995). Genomic comparison of melanoma variants suggested that proteins regulating actin organisation, such as RhoC, were overexpressed in highly metastatic melanoma cells (Clark *et al*, 2000). It has also been shown to regulate tumour progression in various carcinomas (Suwa *et al*, 1998; Clark *et al*, 2000; Kleer *et al*, 2002; Kondo *et al*, 2004). He *et al* (2008) showed that RhoC-siRNA (short-interfering RNA) reduced adhesion to Matrigel invasion and migration of SiHa cells. Studies indicate that a cross-talk exists between RhoC and other signalling molecules such as MAPK and vascular endothelial growth factor (Vegf). RhoC modulates the MAPK pathway and increases Vegf, basic fibroblastic growth factor and interleukins 6 and 8 expression (van Golen *et al*, 2000a, 2002). In another study, RhoC-siRNA inhibited the growth and invasion of gastric carcinoma cells through the PI3K/Akt pathway (Sun *et al*, 2007), and regulated Pyk2 during tumour metastasis (Iiizumi *et al*, 2008).

In the absence of reported mutations of the Notch1 locus, as in leukaemias (Weng *et al*, 2004), the mechanism of Notch activation is likely to involve dysregulated ligand expression or altered patterns of modifiers of the pathway. As phenotypically both Notch and RhoC signalling molecules regulate similar features during tumour progression, we explore the possibility of RhoC being a downstream effector of Notch1. Our results show that RhoC contributes to invasion, EMT, anoikis resistance, tumour growth and angiogenesis in cervical carcinoma. We show that inhibition of Notch1 inactivated the RhoC protein. Using a combination of genetic and molecular tools, we show that Notch1 inactivation can be rescued by RhoC. Constitutively active RhoC (caRhoC) can rescue phenotypic changes associated with siRNA-mediated Notch1 inactivation. Immunohistochemical analysis of archival tissue suggests a correlation between RhoC and Notch1 expression in human cervical carcinoma tissues. The results imply that RhoC is an effector of Notch1.

## Materials and methods

### Cell culture and reagents

CaSki, SiHa and HaCaT cells were cultured in 1 × Dulbecco's modified Eagle's medium (DMEM) containing 10% FBS (foetal bovine serum) and penicillin/streptomycin (100 *μ*g ml^−1^) at 37°C in 5% CO_2_ atmosphere. LY294002, GGTI and DAPT were purchased from Calbiochem (Darmstadt, Germany) and Sigma (Poole, UK). The following plasmid constructs were used: pcDNA3-Neo (Invitrogen, Carlsbad, USA), wild-type RhoC (wtRhoC) and dominant-negative RhoC (dnRhoC) (UMR cDNA Resource Center), mouse caRhoC (gift from Professor SA Baldwin), glutathione *S*-transferase-Rho binding domain (GST-RBD) (gift from Professor A Ridley) and dn-Akt (Rangarajan *et al*, 2001). To generate stable clones, transfected cells were treated with G418 and drug-resistant colonies were selected after RhoC expression analysis. All transfections were carried out with Lipofectamine 2000 (Invitrogen), as specified by the manufacturer.

### RNA interference

Short-interfering RNAs for Notch1 (ID 16704), RhoC (ID 120897) and negative control (−ve siRNA) were purchased from Ambion (Austin, TX, USA). After transfection with 30 pmol of siRNA, using Lipofectamine 2000 (Invitrogen), cells were cultured for 48 h before harvesting for further experiments.

The Notch1 (N1)-shRNA and scrambled shRNA was cloned in GenScript vector (pRNATin-H1.2/Neo) as a *Bam*HI/*Hin*dIII fragment.

N1-shRNA: (5′-GGATCCCACTCGCAGTGGAAGTCATTGATTGATATCCGTCAATGACTTCCACTGCGAGTTTTTTTCCAAAAGCTT) and scramble shRNA: (5′-GGATCCCAAGTCACGCTAGTGATAGAATTTGATATCCGATTCTATCACTAGCGTGACTTTTTTTTCCAAAAGCTT).

### Immunoblotting

Cells washed with cold PBS were incubated with lysis buffer (20 mM Tris–HCl (pH 7.5), 150 mM NaCl, 1% NP-40, 1% (v/v) sodium deoxycholate, 0.1% (w/v) SDS, 50 mM NaF, 1 mM Na_3_VO_4_, 50 *μ*g ml^−1^ PMSF, 1 *μ*g ml^−1^ leupeptin, 1 *μ*g ml^−1^ pepstatin) for 30 min on ice, homogenised with a 23-G needle and centrifuged at 14 000 r.p.m. for 10 min at 4°C. The lysate was resolved using SDS–PAGE, blotted and probed using the appropriate antibody. The primary antibodies used were *β*-actin (Sigma, clone AC-74), Notch1 (Sigma, cat. no. N6786), Vegf (Oncogene, Cambridge, MA, USA, PC-36) and RhoC (Santa Cruz Biotechnology, Inc., CA, USA, cat. no. sc-26481) at appropriate dilutions.

### Immunohistochemistry

Immunohistochemistry was performed on formalin-fixed, paraffin-embedded tissue sections. Briefly, the sections were deparaffinised in xylene, followed by rehydration. After peroxide quenching and antigen retrieval with sodium citrate, the sections were incubated overnight at 4°C with the following antibodies: RhoC (Santa Cruz Biotechnology, Inc., cat. no. sc-26481), Notch1 (Santa Cruz Biotechnology, Inc.), Hif*α* (Santa Cruz Biotechnology, Inc, cat. no. sc-10790), Vegf (Oncogene, PC-36), Akt (Cell Signaling Technology, Beverly, MA, USA, clone 736E11) and anti-mouse CD31 (BD Pharmingen, CA, USA, cat. no. 550274). The sections were then successively incubated with biotinylated secondary antibody (Vector Laboratories, Burlingame, CA, USA) and streptavidin–HRP complex (Vector Laboratories) at room temperature for 40 min each. The colour was developed using DAB (3,3′-diaminobenzidine, Sigma, UK). After counterstaining with haematoxylin, sections were dehydrated, mounted and visualised under a microscope.

Immunoreactivity was considered significant when the characteristic immunostaining was observed in more than 10% of cells. The protein expression was classified as mild (+), moderate (++) and intense (+++) on the basis of the intensity of immunostaining.

### Immunofluorescence

Cells grown on coverslips were fixed with 4% (w/v) paraformaldehyde (PFA) for 10 min at 37°C. The cells were quenched with 50 mM NH_4_Cl and permeabilised with 0.1% (v/v) Triton X-100 in PBS for 5 min. After blocking for 1 h with 0.2% (v/v) fish skin gelatin in PBS, the cells were incubated overnight with the following primary antibodies: anti-fibronectin (Sigma, cat. no. F6140), Notch1 (Santa Cruz Biotechnology), RhoC (Santa Cruz Biotechnology, Inc, cat. no. sc-26481) and Plakoglobin (Sigma, cat. no. P8087), followed by appropriate secondary antibody conjugates. Finally, the coverslips were mounted on microscope slides and imaged using Nikon Inverted Microscope ECLIPSE TE2000-S, Melville, NY, USA.

### Wound-healing assay

Cells cultured to 95% confluence were scratched with a 10-*μ*l pipette tip. Wounds were photographed at 0 h time point, followed by imaging at later time points, ensuing wound closure using a Nikon Inverted Microscope ECLIPSE TE2000-S. The nude area was measured using Image-Pro-Plus (Media Cybernetics, Bethesda, MD, USA) and percentage wound closure was calculated.

### Anoikis assay

Cells were cultured in 1 × DMEM in PolyHEMA-coated dishes, harvested at different time points, fixed with 4% PFA, stained with Hoechst (1 *μ*g ml^−1^; Sigma) and cell death was estimated by counting apoptotic nuclei using the Nikon Inverted Microscope ECLIPSE TE2000-S.

### Soft agar colony formation

Cell lines were transfected with indicated plasmids and cultured for 48 h. They were trypsinised and 2 × 10^5^ cells were mixed in 0.33% agar in DMEM containing 10% FBS and seeded into 35 mm dishes containing 0.5% agar in DMEM. Cells were grown for 21 days and 200 *μ*l of medium was added every third day to the dishes. At the end of 21 days, 10 random fields were selected and colonies were counted using a Nikon Inverted Microscope ECLIPSE TE2000-S.

### Invasion and motility assay

For motility and invasion assay, 0.5 × 10^6^ cells in 1 × DMEM were seeded in the top chamber of uncoated or Matrigel-coated transwell filters with 8 *μ*m pores (BD Biosciences, CA, USA). The bottom chamber contained 1 × DMEM with 10% FBS and served as a chemoattractant. After incubation for 20 h at 37°C, the cells at the bottom of the upper chamber were stained with Hoechst and counted under the Nikon Inverted Microscope ECLIPSE TE2000-S.

### RhoC activity assays

Rho activity assays were performed using the GSTRBD fusion protein, Rhotekin (for Rho activity) (a gift from Professor Anne Ridley, UCL, London, UK). Briefly, glutathione sepharose was used to extract GST-RBD from BL21 cells expressing the GST-RBD fusion protein. Cultured mammalian cells (pharmacologically treated and untreated) were lysed using ice-cold lysis buffer (25 mM Hepes (pH 7.5), 150 mM NaCl, 10 mmol l^−1^ MgCl_2_, 1% NP-40, 1 mM EDTA, 25 mM NaF, 1 mM NaO_4_Va and 10% glycerol along with 2 mmol l^−1^ phenylmethylsulphonyl fluoride. Lysates were clarified, protein content was estimated using a BCA assay and an equal amount of protein was taken for the assay. GST-RBD-coupled sepharose beads were added to the lysates and incubated for 1 h at 4°C to pull down active RhoC. Beads were washed, denatured, separated by electrophoresis and transferred onto a nitrocellulose membrane for subsequent analysis by western blotting.

### MTT assay

An equal number of cells were seeded in 96-well plates and cultured for varying hours. At given time points, the cells were washed with PBS, and 200 *μ*l of MTT (Sigma) working solution (0.5 mg ml^−1^) was added to the wells and incubated at 37°C for 2 h. The media were removed and the converted dye was solubilised in 200 *μ*l isopropanol. The absorbance of the converted dye was measured at a wavelength of 570 nm with background subtraction at 650 nm.

### Tumour formation in nude mice

Cells were grown in 145-mm-diameter dishes to confluence, trypsinised, resuspended in 200 *μ*l of 1 × DMEM and injected subcutaneously into the flanks of N:NIH-Swiss mice. Each mouse (aged between 6 and 8 weeks), were injected with 5 × 10^6^ cells. After 21 days, the mice were killed and the tumour was weighed.

### Measurement of tumour vascularity

To measure vascularity, the tumours were processed for immunohistochemical analysis by fixing the tumour overnight in 4% PFA and cryoprotecting in increasing concentrations of sucrose (10–30%). Tissues were embedded in O.C.T. embedding medium and 4 *μ*m sections were collected. The cryosections were immunostained for RhoC and rat anti-mouse CD31 (BD Pharmingen). Sections were examined by light microscopy under magnifications × 400, and the number of blood vessels from 10 high-power fields was counted using the Nikon Inverted Microscope ECLIPSE TE2000-S.

### *In vitro* tube formation assay

A 50-*μ*l Matrigel bed (60%) was prepared in wells of 96-well plates and was allowed to set for 2 h at 37°C in 5% CO_2_ in a humidified incubator. A total of 7000 human umbilical vascular endothelial cells were seeded per well in EBM2 media (Cambrex, MD, USA). The media was supplemented with 50 *μ*l of conditioned media for the assay and tube formation allowed for 16 h. The tubes were imaged using a Nikon Inverted Microscope ECLIPSE TE2000-S (at 200 × magnification) and the length of the tube was measured as the distance between two nodes using Image-Pro-Plus (Media Cybernetics).

### Statistical analysis

Significance of data has been analysed by Student's *t*-test. The correlation of RhoC expression with cervical carcinoma and other parameters was analysed by the *χ*^2^-test.

## Results

### RhoC drives tumour progression in cervical carcinoma

**Figure 1:**
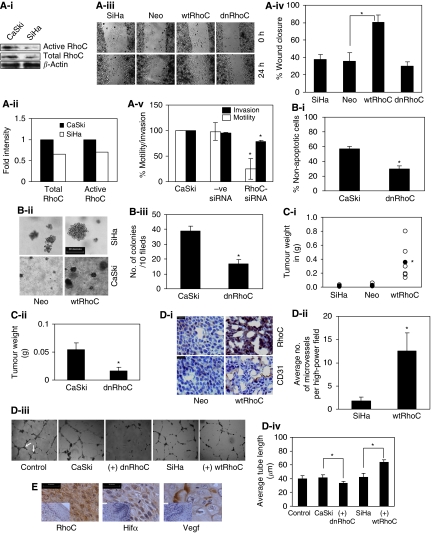
RhoC drives tumour progression in cervical carcinoma. (**A-i**) Cell extracts were analysed for RhoC expression in CaSki and SiHa cells. The SiHa cells show less RhoC expression and activity compared with CaSki cells (*n*=2). (**A-ii**) Graphical presentation of panel Ai. (**A-iii**) Representative images showing wtRhoC expression in SiHa cells increased motility in a wound-healing assay (magnification × 40). (**A-iv**) Graphical presentation of panel A-iii showing faster wound closure by SiHa-wtRhoC compared with SiHa-Neo (*P*<0.05; *n*=4). (**A-v**) RhoC-siRNA reduced motility (*P*<0.01) and invasion (*P*<0.02) of CaSki cells, –ve siRNA did not alter motility and invasion of CaSki cells. CaSki cells were transfected with siRNA for 48 h, cells were trypsinised and seeded for a transwell migration assay (*n*=3). (**B-i**) Graphical presentation of the effect of dnRhoC resistance to anoikis. Expression of dnRhoC led to decreased CaSki cell survival (30% non-apoptotic cells; *P*<0.001) cultured for 7 h under anoikis (*n*=3). (**B-ii**) CaSki and SiHa cells formed large colonies upon wtRhoC overexpression compared with parental cells. Cells were transiently transfected with wtRhoC, cultured for 48 h and were seeded for a clonogenic assay (*n*=3). (**B-iii**) Graphical representation of the effect of dnRhoC on colony formation. The expression of dnRhoC resulted in lesser number of colonies (16/10 fields; *P*<0.003) compared with parental CaSki cells (*n*=3). (**C-i**) Overexpression of wtRhoC in SiHa cells resulted in tumours 15-folds heavier (*P*<0.02) than SiHa tumours. The black-filled circles indicate the mean tumour weight for each tumour type (*n*=6). The empty circles indicate individual tumour weights (*n*=6). (**C-ii**) Expression of dnRhoC in CaSki cells resulted in smaller tumours, average weight 0.04 g (*P*<0.019) in xenograft assays in nude mice (*n*=3). (**D-i**) Xenograft cryosections were stained for RhoC and CD31. Representative images suggest increased RhoC and microvessel-specific CD31 expression in SiHa-wtRhoC tumours. (**D-ii**) Graphical representation of microvessel density in SiHa-wtRhoC xenografts. Expression of wtRhoC formed more blood vessels, ∼12.5 (*P*<0.009) microvessels per high-power field compared with SiHa tumours. The lumen with CD31 expression was counted in 10 high-power fields from at least four different sections. (**D-iii**) Illustrative images representing the effect of RhoC on tube formation by human umbilical vascular endothelial cells (HUVECs). A total of 7000 cells were seeded in 60% Matrigel (BD Biosciences) and cultured for 16 h with indicated conditioned medium. The distance between the two arrows indicates the length of the tubes (magnification × 200). (**D-iv**) Ectopic expression of RhoC and its mutant altered *in vitro* tube formation by HUVECs in Matrigel. Conditioned media from CaSki-dnRhoC and SiHa-wtRhoC, respectively, decreased and increased tube formation (*P*<0.001; *n*=3). (**E**) Cervical carcinoma tissue sections immunohistochemically assessed for expression of RhoC, Hif*α* and Vegf in serial sections of same tumour sample (scale bar=30 *μ*m). Corresponding normal tissue section images presented as inset. ^*^Significant difference between the data set.

The transition from *in situ* carcinoma to metastatic tumour in solid tumours involves motility, invasion, anoikis resistance and sustained growth. To assess the contribution of RhoC to invasion and metastasis, we used cervical carcinoma-derived CaSki and SiHa cells. Figure 1A-i and A-ii shows the expression levels of RhoC in CaSki and SiHa cells, with higher total RhoC and higher active RhoC levels in CaSki cells compared with SiHa cells. This phenotype was complemented in SiHa cells by stably expressing wtRhoC in stable clones (Supplementary Figure 1A). Dominant-negative RhoC was also stably expressed in CaSki and SiHa cells.

To assess the contribution of RhoC to motility, we used stable clones of SiHa cells expressing wtRhoC and dnRhoC in a wound-healing assay. SiHa-wtRhoC cells closed 80% (*P*<0.05) of the wound area as compared with 35% by SiHa Neo cells (expressing empty vector) in 24 h (Figure 1A-iii and iv). The effect of RhoC depletion on motility and invasion of CaSki cells in a transwell chamber assay is shown in Figure 1A-v. Compared with control siRNA (−ve siRNA), RhoC-siRNA treatment resulted in a 75% (*P*<0.01) and 45% (*P*<0.02) decrease in motility and invasion, respectively, of CaSki cells. The specificity of siRNA activity has been shown in Supplementary Figure 1B. Immunofluorescent analysis of RhoC expression in squamous cervical carcinoma (SCC) tissues from patients shows the presence of RhoC-expressing cells in adjoining blood vessels (Supplementary Figure 1C), suggesting that RhoC is involved in metastasis.

Metastatic cells need to resist anoikis in blood vessels to re-grow as metastases at a distant site. Figure 1B-i shows that a reduction in RhoC protects cells from anoikis. When cultured for 7 h under anoikis, there were 30% (*P*<0.001) non-apoptotic CaSki-dnRhoC cells as compared with 57% non-apoptotic CaSki cells. Similarly, wtRhoC protected SiHa cells maximally from anoikis for 48 h (Supplementary Figure 1D).

CaSki and SiHa cells transiently expressing wtRhoC formed bigger colonies (Figure 1B-ii), whereas dnRhoC resulted in a lesser number of colonies (Figure 1B-iii) in a clonogenic assay. The number of colonies formed by the CaSki-dnRhoC clone (16 colonies/10 fields) was significantly less compared with CaSki cells forming 39 colonies/10 fields (*P*<0.003).

To determine the contribution of RhoC to tumour growth, we used (5 × 10^6^ cells) stable clones of CaSki and SiHa cells expressing dnRhoC and wtRhoC, respectively, and generated xenografts in nude mice over a period of 21 days. As illustrated in Figure 1C-i and ii, the ectopic expression of RhoC and its mutant altered the tumour growth properties of CaSki and SiHa cells. SiHa-wtRhoC cells formed larger tumours (mean weight 353 mg; *P*<0.02) compared with SiHa cells (16 mg). Similarly, CaSki-dnRhoC cells formed tumours that were significantly smaller (0.016 g; *P*<0.047) than those formed by CaSki cells (0.054 g). Interestingly, the tumours formed by SiHa-wtRhoC cells were more angiogenic, with a high microvessel density of 12.5 (*P*<0.009) microvessels, as opposed to 1.75 in SiHa tumours (Figure 1D-i and ii; Supplementary Figure 2A). An immunohistochemical analysis for CD31 and RhoC on xenograft sections suggests CD31 localisation at the microvessels. We measured the effect of RhoC on *in vitro* tube formation using human umbilical vascular endothelial cells. As illustrated in Figure 1D-iii and iv, conditioned media derived from cultures of CaSki, CaSki-dnRhoC, SiHa and SiHa-wtRhoC cells were used to test the effect. There was significant loss in tube formation and a resultant reduction in tube length with CaSki-dnRhoC-derived media (33.66 *μ*m; *P*<0.0001) as compared with CaSki (41.33 *μ*m) media, whereas wtRhoC in SiHa cells enhanced the tube length (63.45 *μ*m; *P*<0.001) as compared with SiHa cells. We extended these observations to clinical sections and assessed the expression of RhoC, Hif*α* and Vegf in matched cervical samples. Representative images suggest an expression of RhoC, Hif*α* and Vegf in the same area of the tumour (Figure 1E) with negligible immunodetection in normal cervix tissues suggesting a co-expression of these molecules. Moreover, a change was observed in Vegf expression because of RhoC expression. Supplementary Figure 2B shows that ectopic expressions of wtRhoC and dnRhoC resulted in elevated and reduced Vegf levels, respectively, in SiHa and CaSki cells.

### Do Notch1 and RhoC regulate similar function during tumour progression?

Notch1 cooperates with various signalling pathways to promote tumour progression, including NF-*κ*B, VEGF, TGF-*β* and p53 (Nair *et al*, 2003; Masuda *et al*, 2005; Wang *et al*, 2006). As both Notch1 and RhoC are involved in tumour progression and metastasis, we investigated whether the two signalling molecules regulate similar functions during cervical tumour progression. Treatment of SiHa and CaSki cells with DAPT (presenilin-dependent gamma-secretase inhibitor that blocks proteolytic processing of Notch1) blocked wound healing in CaSki cells but had no effect in SiHa cells (Supplementary Figure 2C). Furthermore, CaSki cells are phenotypically similar to most cervical cancers with regard to its expression of Jagged1, features of activated Notch1 and PI3K signalling and dependence on Notch1 signalling for growth and survival (Veeraraghavalu *et al*, 2005). We thus used CaSki cells to assess the relationship between RhoC and Notch1.

**Figure 2:**
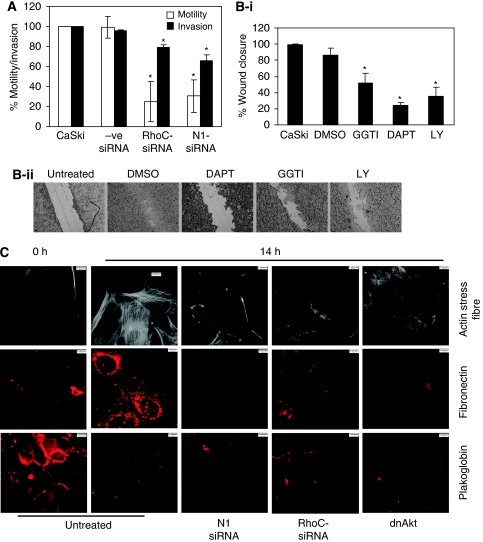
Does Notch1 and RhoC regulate similar function during tumour progression. (**A**) RhoC-siRNA and N1-siRNA reduced motility (*P*<0.05) and invasion (*P*<0.05) of CaSki cells. −ve siRNA did not alter motility and invasion of CaSki cells. CaSki cells were transfected with siRNA for 48 h, cells trypsinised and seeded for a transwell migration assay (*n*=3). (**B-i**) Representative images showing DAPT, LY294002 (LY) and GGTI decreased wound healing of CaSki cells at 14 h. DAPT (*P*<0.0002), GGTI (*P*<0.03) and LY (*P*<0.05) significantly reduced motility during wound healing (*n*=3). (**B-ii**) Representative images of wound healing of CaSki cells in the presence of DAPT, GGTI and LY. (**C**) Representative images of changes in actin organisation following EMT during wound healing. Cells were transfected with the siRNA as mentioned and dnAkt. They were cultured for 48 h and were scratched with a 10-*μ*l pipette tip. The cells were allowed to close the wound for 14 h, fixed with 3% paraformaldehyde and immunostained for actin stress fibres, fibronectin and plakoglobin expression following EMT during wound healing. Each experiment has been carried out at least three times.

Depletion of Notch1 and RhoC with siRNA against Notch1 (N1-siRNA) and RhoC (RhoC-siRNA) inhibited motility and invasion in CaSki cells (Figure 2A). The motility of CaSki cells reduced to 25% (*P*<0.01) and 30% (*P*<0.01) for N1-siRNA- and RhoC-siRNA-transfected cells. There were 78% (*P*<0.01) and 66% (*P*<0.01) invading RhoC and Notch1 knockout cells compared with CaSki cells. The effect of N1-siRNA on Notch1 levels has been shown (Supplementary Figure 2D). Concurrently, in a wound-healing assay, inhibition of Notch1 with DAPT (40 *μ*M), and that of PI3K with chemical inhibitor LY294002 (LY; 20 *μ*M) and RhoGTPase inhibitor (geranylgeranyl transferase inhibitor (GGTI); 10 *μ*M) for 14 h caused reduced wound healing with 24, 52 and 35% (*P*<0.03) wound closure, respectively, compared with DMSO-treated cells. Treatment of CaSki cells with the above inhibitors also resulted in RhoC localisation changes (Supplementary Figure 2E).

As reported earlier, the Notch1 ligand, Jagged1, generates EMT response in CaSki cells in a Notch1-PI3K-dependent but CBF-independent manner (Veeraraghavalu *et al*, 2005). In this study, CaSki cells transfected with N1-siRNA, RhoC-siRNA and dominant-negative Akt (dn-Akt) were subjected to wound healing for 14 h and assessed for fibronectin, plakoglobin and actin stress fibre organisation. With the progression of wound healing, there was elevated fibronectin expression and actin stress fibre formation in untreated CaSki cells at 14 h (Figure 2C); however, N1-siRNA, RhoC-siRNA and dnAkt prevented fibronectin expression and actin stress fibre formation in cells adjoining the wound site. Control cells at the 0 h time point neither showed fibronectin expression nor had actin stress fibre formation. Plakoglobin expression was lost in untreated cells during wound healing at 14 h but contrary to reversal of fibronectin expression, plakoglobin expression showed no reversal with inhibitors. The data collectively suggest that RhoC phenocopies the effect of Notch1 in EMT and invasion.

### Is RhoC function regulated by Notch1?

**Figure 3:**
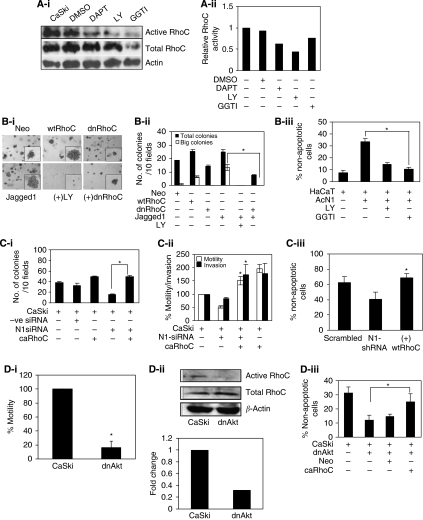
Is RhoC function regulated by Notch1. (**A-i**) RBD-GST pull down protein extracts of CaSki cells treated with DAPT, LY and GGTI were assessed for levels of active RhoC. The crude protein extracts were immunoblotted for total RhoC and *β*-actin (indicating loading control). There was decrease in active RhoC levels in DAPT- and LY-treated cells compared untreated cells. (**A-ii**) Graphical presentation of panel A-i showing relative RhoC activation (active RhoC/total RhoC). (**B-i**) Expression of dnRhoC suppresses the Jagged1-mediated increased colony formation of SiHa cells. Co-expression of dnRhoC in Jagged1-transfected SiHa cells resulted in reduced colony formation (*P*<0.005; *n*=3). (**B-ii**) Graphical representation of panel B-i. (**B-iii**) RhoGTPase inhibition blocks enhanced anoikis survival of HaCaT-ACN1 cells. Following GGTI (10 *μ*m) treatment, only 10% cell survival was observed compared with 33% non-apoptotic HaCaT-ACN1 cells (*P*<0.002; *n*=3). (**C-i**) Notch1 depletion phenotype was rescued by caRhoC in a clonogenic assay. N1-siRNA-transfected CaSki cells formed 15 colonies/10 fields while co-expression of caRhoC resulted in 49 colonies/10 fields (*P*<0.0001). CaSki cells were transfected with Notch1 siRNA (30 pmol ml^−1^) for 48 h. Cells were trypsinised and 10 000 cells were seeded for a clonogenic assay (*n*=3). (**C-ii**) Notch1 depletion phenotype was rescued by caRhoC in a transwell migration assay. N1-siRNA-treated cells resulted in less invasion (82%) and motility (51%) compared with CaSki cells, whereas complementation with caRhoC resulted in 173% invasion (*P*<0.04) and 151% motility (*P*<0.005) comparable with CaSki cells transfected with caRhoC alone (*n*=3). (**C-iii**) Notch1 depletion phenotype was rescued by caRhoC in an anoikis resistance assay. N1 shRNA resulted in reduced cell survival (40%) compared with CaSki cells. Complementation with caRhoC resulted in 68% live cells (*P*<0.005; *n*=3). (**D-i**) CaSki-dnAkt cells were less motile (16%; *P*<0.003) compared with CaSki cells in a transwell migration assay (*n*=3). (**D-ii**) Extracts from CaSki and CaSki-dnAkt were assayed for active RhoC and total RhoC levels. There was reduced RhoC activity in CaSki-dnAkt cells. (**D-iii**) Akt inactivation phenotype was rescued by caRhoC in an anoikis resistance assay. Complementation of CaSki-dnAkt cells with caRhoC resulted in increased cell survival (25% non-apoptotic cells; *P*<0.01) compared with 12% CaSki-dnAkt cells (*n*=3). ^*^Significant difference between the data set.

The phenotypic similarity between Notch1 and RhoC suggests that RhoC may be regulated by Notch1. To assess this relationship, we used GST-RBD (Taylor *et al*, 2001) to pull down active RhoC-GTP after treating the cells with the Notch and PI3K inhibitors. Figure 3A illustrates that the RhoC activation level is decreased after Notch1 inhibition. The same results were obtained after PI3K inhibition, indicating that RhoC, which phenocopies the Notch1 effect on EMT, is regulated by the latter. SiHa cells that lack Jagged1 were used to assess the regulation of RhoC by Notch1. As shown in Figure 3B-i and -ii, overexpression of wtRhoC and Jagged1 in SiHa cells formed big (diameter >30 mm) colonies (6 and 13/10 fields, respectively) in a clonogenic assay. Co-expression of dnRhoC in Jagged1-expressing SiHa cells led to the abolition of big colonies (*P*<0.005). We also show that inhibition of PI3K resulted in the abolition of colonies formed by Jagged1 (Figure 3B-i). This is in agreement with an earlier report that PI3K mediates Jagged1-mediated EMT changes (Veeraraghavalu *et al*, 2005). The Notch1 expression in HaCaT cells (HaCaT-ACN1) enhances anoikis resistance through the PKB/Akt pathway (Rangarajan *et al*, 2001). In this study, we show that GGTI treatment resulted in reduced anoikis resistance of HaCaT-ACN1 cells. HaCaT-ACN1 cells showed 33% non-apoptotic cells at 14 h, which reduced to 10% (*P*<0.002) on treatment with GGTI (Figure 3B-iii).

Rescue of RNAi-mediated loss-of-function phenotype by expressing caRhoC, which is refractory to upstream signalling events, was used to further corroborate RhoC as a downstream target of Notch1. Ectopic expression of caRhoC in CaSki cells with depleted Notch1, by N1-siRNA or Notch1 shRNA (N1-shRNA), rescued the loss-of-function phenotype of Notch1. As shown in Figure 3C-i, the N1-siRNA expression in CaSki cells reduced colonies to 15/10 fields compared with 32 control colonies. Expectedly, co-expression of caRhoC resulted in significantly increased colonies (49/10 fields; *P*<0.0001). Similarly, caRhoC also rescued the inhibition of the motility and invasion of CaSki cells because of N1-siRNA expression. Co-expression of caRhoC resulted in a significant increase in motility (151%; *P*<0.005) and invasion (173%; *P*<0.04) compared with N1-siRNA (51% motility and 82% invasion)-transfected cells (Figure 3C-ii). Co-expression of caRhoC in CaSki cells carrying N1-shRNA also enhanced anoikis resistance (68% non-apoptotic cells; *P*<0.005) compared with N1-shRNA cells (40% non-apoptotic cells). The specificity of N1-shRNA and N1-siRNA has been shown in Supplementary Figure 2D.

As RhoC inhibition phenocopied the Notch1 and PI3K inhibitory phenotype, we used CaSki-dnAkt cells (Rangarajan *et al*, 2001) to confirm the effect of PI3K inhibition of RhoC activity in Figure 3A. Expression of dnAkt in CaSki cells caused decreased motility and enhanced anoikis. As shown in Figure 3D-i, there was significant reduction in the motility of CaSki-dnAkt cells (16%; *P*<0.003) compared with CaSki cells. RhoC activity as assessed by RBD-GST binding suggested a decreased activity of RhoC in CaSki-dnAkt cells (Figure 3D-ii). Subsequently, as expected, overexpression of caRhoC in CaSki-dnAkt cells generated enhanced anoikis resistance (25% non-apoptotic cells; *P*<0.01) compared with 12% non-apoptotic CaSki-dnAkt cells in 14 h. These results convincingly position RhoC as a downstream effector of Notch1 and PI3K signalling in cervical carcinoma cells.

### RhoC expression correlates with Notch1 expression in cervical tumour progression

**Figure 4:**
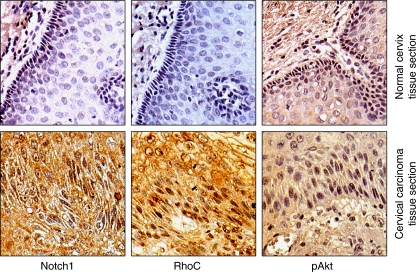
RhoC expression correlates with Notch1 expression in cervical tumour progression. Immunodetection of Notch1, RhoC and pAkt in cervical carcinoma. Immunohistochemical analysis was carried out on paraffin-embedded cervical carcinoma tissue sections using anti-Notch1, anti-RhoC and anti-pAkt antibodies (magnification × 200).

To further corroborate our findings in a clinical setting, we examined the expression of RhoC and its relationship with Notch1 in archival cervical carcinoma specimens using immunohistochemical technique. The specimens were randomly selected and the expression of Notch1, RhoC and pAkt was assessed in them. Our results suggest RhoC is strongly expressed in SCC tissues (Figure 4A). There was significant difference in the extent of cellular positivity and intensity of RhoC expression across the grades (*P*<0.007). Although cytoplasmic expression of RhoC was observed in all the cases across all grades of tissues, the percentage of positive cases increased across the tumour grades. Only 5 out of 12 (41%) normal cervical tissues showed RhoC expression compared with 21 out of 24 (87%) SCC tissues. Of the 87% cases, 65% had mild, 14% had moderate and 8% had intense RhoC expression.

Correlation of RhoC with Notch1 and pAkt was assessed on serial sections of SCC tissues and all the molecules were found to be expressed in the same areas of the various tumour sections (Figure 4B). Importantly, RhoC expression in the SCC specimens showed significant correlation with Notch1 expression (*P*<0.006) and pAkt expression (*P*<0.006). Out of 17 SCC specimens, RhoC was expressed in 14 specimens, whereas Notch1 and pAkt expression was observed in all 17 specimens. These data collectively suggest a correlation between RhoC, Notch1 and pAkt. We thus propose RhoC to be a downstream effector of Notch1 in cervical tumour progression.

## Discussion

Carcinoma progression is a multi-step process engaging various signalling pathways. There are surmounting evidences elucidating the contribution of two such pathways including Notch1 and RhoC to carcinoma progression. Notch1 has been shown to exert regulations on tumour growth, invasion, metastasis and angiogenesis in various carcinomas. Increased expression of RhoC in metastatic melanoma cells in comparison with the poorly metastatic counterparts (Clark *et al*, 2000) was the advent of studies implicating RhoC in carcinoma progression especially metastasis.

In accordance with its contribution to motility and invasion of several cancers (van Golen *et al*, 2000b; He *et al*, 2008), our data show that RhoC contributes to tumour progression by affecting invasion, anoikis resistance, tumour growth and angiogenesis in cervical carcinoma cell lines. We show that RhoC drives tumour progression by regulating invasion, metastasis, tumour growth and angiogenesis (Figure 1). We find increased vasculature associated with increased RhoC expression in xenografts grown from SiHa cells. Immunohistochemical assessment of archival tissue sections showed expression of RhoC, Vegf and Hif*α* in the same areas of tumour sections. Immunofluorescent analysis of paraffin-embedded SCC tissue sections for RhoC expression showed the presence of invading cells in the adjoining blood vessels and at the periphery of the tumour, underlining the importance of RhoC to invasion.

We previously proposed that Notch1 regulates EMT response in the cervical epithelial cell line, CaSki (Veeraraghavalu *et al*, 2005). In this study, we show that RhoC regulates EMT during tumour progression in a manner similar to Notch1. Inactivation of RhoC resulted in similar phenotypic changes, as associated with the effect of Notch and PI3K inhibition of EMT (Figure 2). This finding parallels that of Hutchison *et al* (2009), in which RhoC has been shown to regulate stress fibre formation during EMT. However, we also show that RhoC regulates fibronectin expression during EMT changes. Considering that both Notch1 and RhoC regulate similar features during tumour progression, we explored the possibility of cross-talk between Notch1 and RhoC. Biochemical assays suggest that Notch1 regulates RhoC activity in CaSki cells. In this study, we show that inhibition of Notch and PI3K with pharmacological agents resulted in reduced RhoC activity in CaSki cells (Figure 3), with no effect on total RhoC levels. When SiHa cell-overexpressing Jagged1 was complemented with dnRhoC, there was reduced colony formation. Concomitantly, HaCaT-ACN1 cells (Rangarajan *et al*, 2001) when treated with GGTI resulted in reduced anoikis-resistant cells. Consistent with the observation that Notch1 signalling generates anoikis to resistance in keratinocytes and CaSki cells (Rangarajan *et al*, 2001; Veeraraghavalu *et al*, 2005), we find that complementation of Notch1-depleted CaSki cells with caRhoC resulted in increased resistance to anoikis. In parallel, CaSki cells, expressing Notch1 siRNA or shRNA, when complemented with caRhoC formed more colonies in a colony formation assay (Figure 3). Dominant-negative Akt also reduced RhoC activity in CaSki cells. When CaSki-dnAkt cells were complemented with caRhoC, there was resultant increase in non-apoptotic cells. However, it should be noted that RhoC promotes invasion of human melanoma cells in a PI3K/Akt-dependent manner and promotes metastasis of prostrate cancer cells by activation of Pyk2, which regulates Akt phosphorylation (Ruth *et al*, 2006; Iiizumi *et al*, 2008).

Using immunohistochemical techniques, we show that there is expression of both Notch1 and RhoC in the same areas of cervical carcinoma tissue sections with significant correlation while there was negligible expression of the proteins in the normal cervical tissue sections (Figure 4). We observed that Notch1 activation and activated PI3K/Akt regulate RhoC activity in CaSki cells; but we cannot rule out the role of other signalling pathways regulating RhoC activity. SiHa cells, for example, lack Jagged1-activated Notch1 signalling (Veeraraghavalu *et al*, 2005), yet they exhibit RhoC activity, which may be regulated by another signalling pathway. Notch1, which has no reported mutation in solid tumours, may likely use downstream effectors to execute its functions in tumour progression. We thus propose RhoC as downstream effector of Notch1 during tumour progression. Interestingly, RhoC is not imperative for embryogenesis but is important for metastasis alone (Hakem *et al*, 2005), and thus it can serve as a good therapeutic target. Our data re-iterates the role of Notch1 in carcinoma progression and places RhoC as its downstream target.
