# Supplementary figures and images for: Notch1 regulates the functional contribution of RhoC to cervical carcinoma progression
Source: Br J Cancer. 2009 Dec 1;102(1):196–205. doi: 10.1038/sj.bjc.6605451 (PMC2813755; doi:10.1038/sj.bjc.6605451)

# Supplementary figure 1

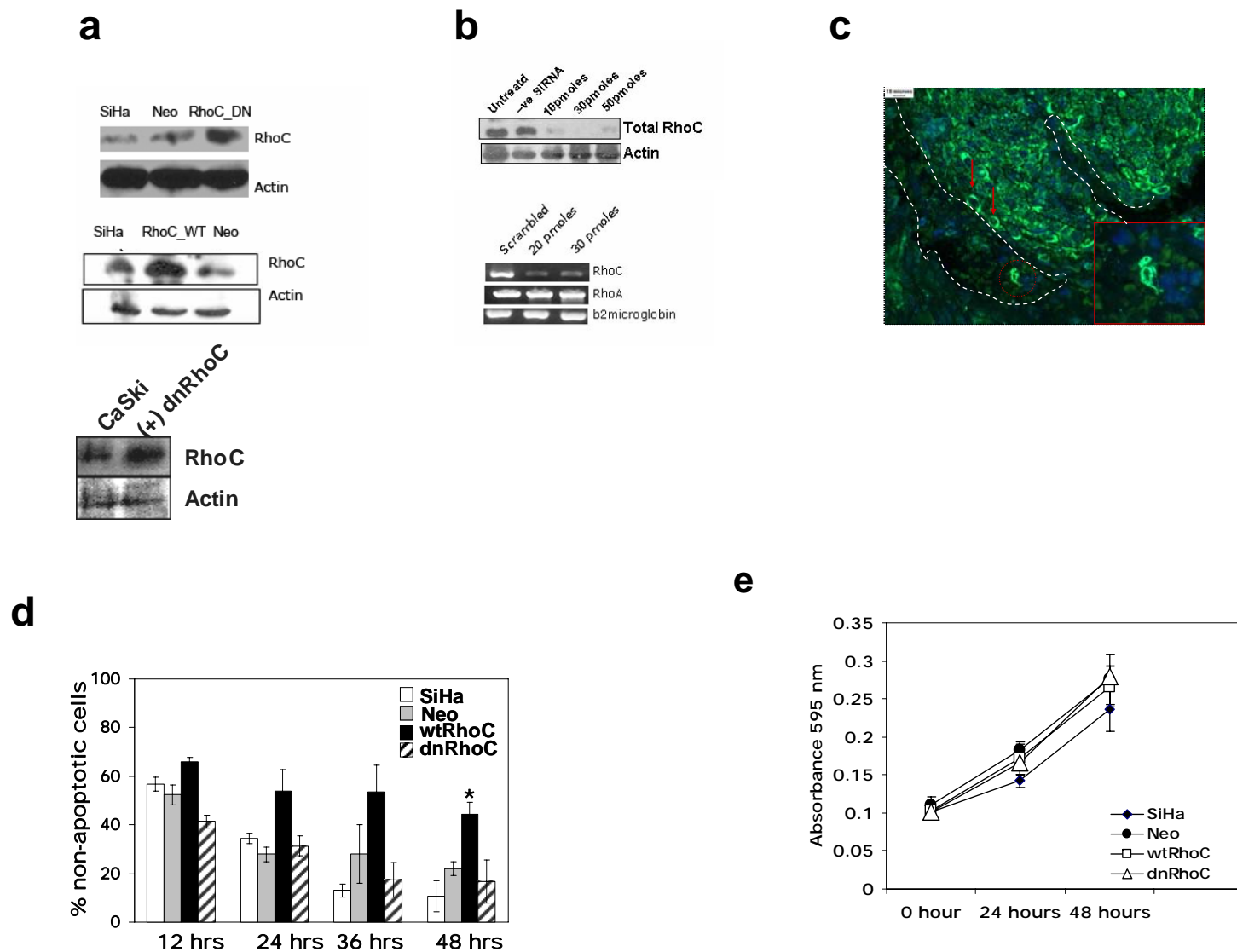

Supplementary figure 2

**a**

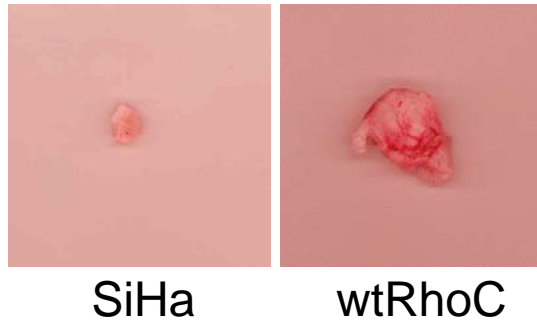

**b-i**

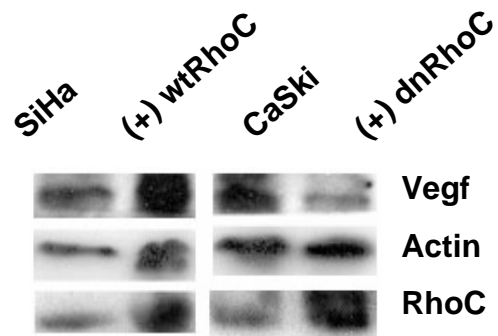

**b-ii**

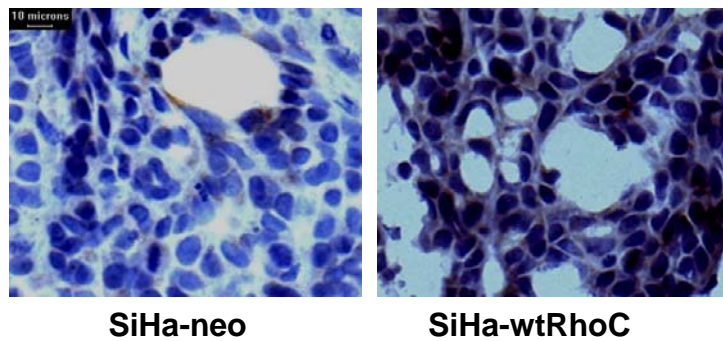

**c**

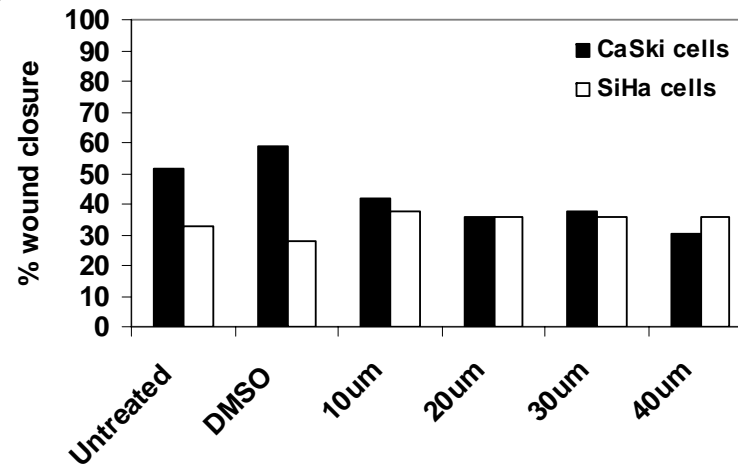

**d**

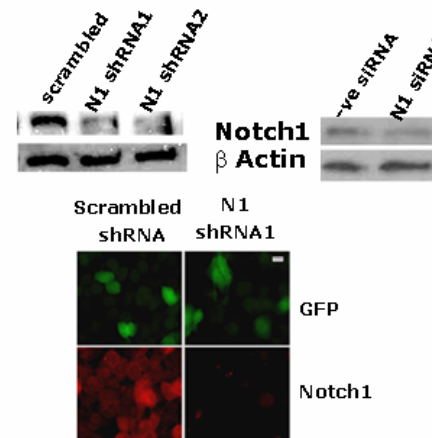

**e**

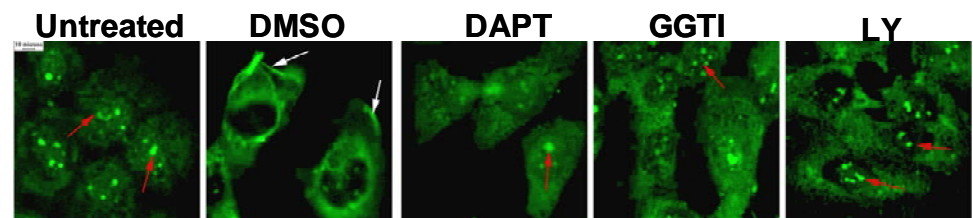

Supplement: Supplementary Figures 1 and 2 [file 6605451x1.pdf]
